# Treatment Switch in Poor Responders with Locally Advanced Gastric Cancer After Neoadjuvant Chemotherapy

**DOI:** 10.1245/s10434-021-10087-x

**Published:** 2021-07-29

**Authors:** Zining Liu, Yinkui Wang, Fei Shan, Xiangji Ying, Yan Zhang, Shuangxi Li, Yongning Jia, Rulin Miao, Kan Xue, Zhemin Li, Ziyu Li, Jiafu Ji

**Affiliations:** Key Laboratory of Carcinogenesis and Translational Research, Ministry of Education/Beijing), Gastrointestinal Cancer Center, Peking University Cancer Hospital and Institute, Beijing, China

## Abstract

**Background:**

Among locally advanced gastric cancer (LAGC) patients, poor response to initial neoadjuvant chemotherapy (NAC) is associated with unfavorable outcomes; however, changing the postoperative therapy regimen in this group of patients is unclear. We compared the poor responders who continued the original protocols with that of patients who switched treatment after NAC plus D2 gastrectomy.

**Methods:**

Our study included LAGC patients who achieved tumor regression grade 3 according to the American Joint Committee on Cancer/College of American Pathologists system, after NAC, between December 2006 and December 2017 at our institution. Outcomes were overall survival (OS), progression-free survival (PFS), and adverse events during postoperative treatment. The propensity score matching method was used to match patients.

**Results:**

Overall, 160 patients were enrolled in the final analysis set, including 21 switched cases and 139 non-switched cases. A 1:2 matched cohort (21 switching vs. 42 non-switching) was generated to eliminate all confounding factors. No statistical differences were observed in OS and PFS, either in the whole patients (OS: log-rank *p* = 0.804; PFS: log-rank *p* = 0.943) or in the matched cohort (OS: log-rank *p* = 0.907; PFS: log-rank *p* = 0.670) between the two groups. Patients with changed regimens had a significantly higher rate of peripheral neurotoxicity (*p* = 0.045). Contrarily, a lower rate of overall adverse events was observed in the non-switching group with marginal significance (*p* = 0.069).

**Conclusion:**

Adjusting to a non-cross-resistant regimen only by post-NAC pathological evaluation may not be sufficient for designing an effective treatment route for LAGC poor responders. Treatment change required a more scrutinized clinical track, which involved a multifaceted assessment.

**Supplementary Information:**

The online version contains supplementary material available at 10.1245/s10434-021-10087-x.

Perioperative chemotherapy (PEC) and neoadjuvant chemotherapy (NAC) have been broadly practiced in patients with locally advanced gastric cancer (LAGC) for over 10 years in Europe, North America, and, most recently, China.^1^–^3^ These regions all have one feature in common: over two-thirds of patients are detected at an advanced stage of cancer during their first visit.^4^ For most LAGC patients, NAC improves tumor resectability and reinforces the effects of therapeutic surgery.^5^

The effects of NAC on a tumor can be histopathologically evaluated using pathological tumor regression grading (TRG) systems. Generally, patients with a poor pathological response after NAC are usually graded with a higher post-therapy pathological (yp) TNM stage as a result of lower chemosensitivity leading to significantly lower survival outcomes.^6^–^8^ Despite the application of several different standards for TRG assessment, most of these criteria achieved similar predictive values.^9^

However, although the PEC and NAC protocols have been updated over the years, a large proportion of patients respond unsatisfactorily to NAC. The rate of poor responders (Mandard TRG 4–5) in the MAGIC, OE05, and FLOT4 trials was 43%, 80%, and 38%, respectively.^10^–^12^ Therefore, current advances in PEC and NAC, including searching for more effective protocols, reducing toxicity, standardizing dosage, and treatment duration would benefit responders but may not significantly impact outcomes for those who are inherently less chemosensitive. On the other hand, current guidelines for NAC do not have clear recommendations for regimen adaptation when a poor post-NAC response is observed. The restricted available drug selections reduce the merit of NAC in LAGC.^13^–^15^ Few studies have evaluated if adjuvant therapy should be adapted to the pathological response.^16^

The current study investigates the efficacy of a non-cross-resistant drug for LAGC patients who were evaluated pathologically as poor responders after NAC treatment. A propensity score matching (PSM) analysis was utilized to reduce potentially confusing factors and enable the comparison of survival outcomes.

## Methods

### Patients

**Fig. 1:**
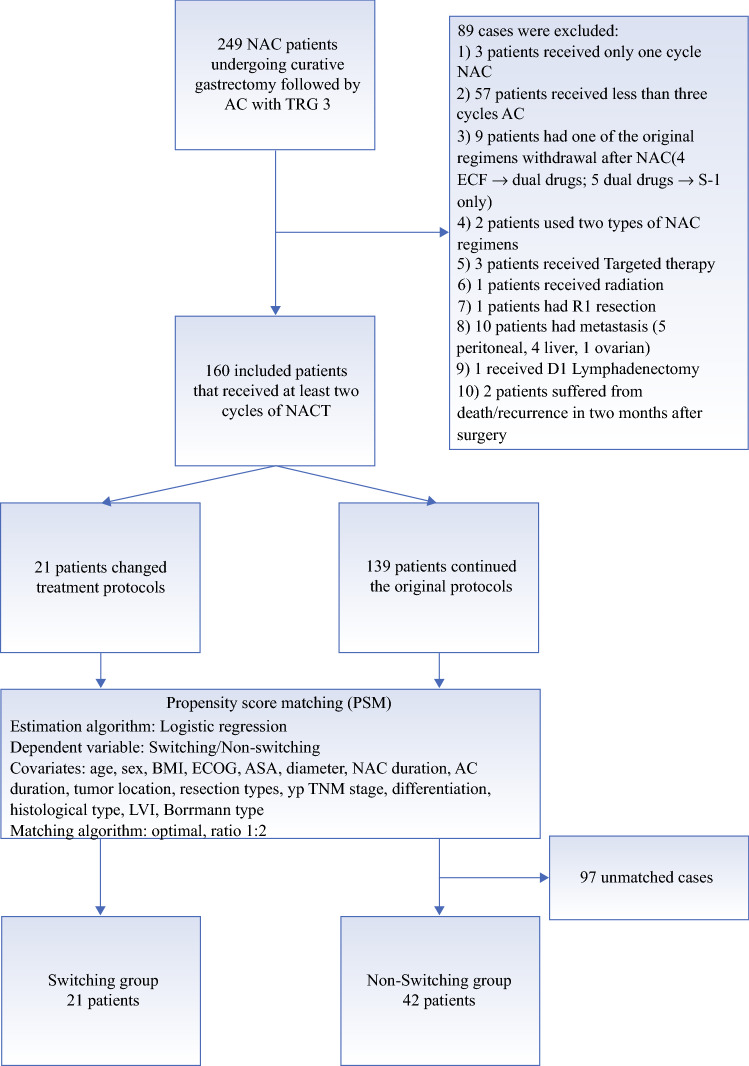
Patient enrolment and propensity score matching process. *NAC* neoadjuvant chemotherapy, *AC* adjuvant chemotherapy, *TRG* tumor regression grade, *ECF* epirubicin, cisplatin, and 5-fluorouracil, *BMI* body mass index, *ECOG* Eastern Cooperative Oncology Group, *ASA* American Society of Anesthesiologists, *LVI* lymphovascular invasion

Data from a prospective database of all patients who started NAC at the Peking University Cancer Hospital and Institute were searched between 1 December 2006 and 1 December 2017. Study inclusion criteria included (1) a proven diagnosis of gastric adenocarcinoma with poor response according to postoperative pathology using the American Joint Committee on Cancer/College of American Pathologists (AJCC/CAP) system;^8^ (2) no signs of distant metastasis at first visit; (3) patients had undergone adjuvant chemotherapy (AC) after surgery; and (4) curative gastrectomy was performed. The exclusion criteria were (1) patients received fewer than two cycles of NAC or fewer than three cycles of AC; (2) patients received reduced regimens of AC compared with NAC; (3) patients used more than one NAC treatment protocol; (4) patients received radiotherapy, targeted therapy, or immune therapy before relapse; (5) patients received hyperthermia intraperitoneal chemotherapy; (6) patients with R1/R2 resection, or suspected of having metastasis or recurrence before completion of three cycles of postoperative treatment; (7) patients with D0/D1/D1 plus lymphadenectomy; and (8) death within 3 months following curative surgery (see Fig. 1).

### Regimen and Radical Surgery

The determination of clinical stages, design for treatment route, preoperative assessment, and prompt intervention for adverse events (AEs) were managed by the multidisciplinary team (MDT). Clinical stages were defined by abdominal computed tomography (CT) scan and/or endoscopic ultrasound (EUS) and/or pretherapeutic laparoscopic exploration. All patients used paclitaxel plus 5-fluorouracil arms or cisplatin plus 5-fluorouracil arms as preoperative or postoperative treatments. Regimens, including oxaliplatin plus S-1 (SOX), oxaliplatin plus capecitabine (CapeOX), oxaliplatin plus 5-fluorouracil/leucovorin (FOLFOX), cisplatin plus S-1 (CS), paclitaxel plus capecitabine (PX), and paclitaxel plus S-1 (PS), are summarized in electronic supplementary Table S1. To assess the duration of treatment, 2-week protocols were recalculated as 3-week protocols to enable direct comparisons between regimens. Dosage reductions or cessation occurred if severe AEs were observed during chemotherapy, as determined by the MDT members. After two to three cycles of chemotherapy, the antitumor effect was evaluated using CT scans. The therapy was prematurely terminated in cases of disease progression, with a curative gastrectomy being immediately performed. Otherwise, gastrectomy or continued NAC was considered after obtaining informed consent and approval from each patient. Subtotal or total gastrectomy plus D2 lymphadenectomy was performed according to the Japanese Gastric Cancer Association (JGCA) guidelines.^17^

### Data Collection

Patient characteristics, including age, body mass index (BMI), sex, American Society of Anesthesiologists (ASA) score, Eastern Cooperative Oncology Group (ECOG) performance status, tumor location, tumor diameter (on short axis), differentiation grade, vascular involvement, post-therapy pathological (yp) TNM stage according to the 8th AJCC guidelines, type of gastrectomy, severe complications classified as Clavien–Dindo grades III–IV, Borrmann type, duration of NAC, and duration of AC were all recorded.^18^,^19^ Chemotherapy-related toxicities observed were systematically and routinely documented according to the Common Terminology Criteria for Adverse Events (CTCAE) version 4.0, including leukopenia, anemia, thrombocytopenia, hepatotoxicity, digestive tract disorders (including nausea, vomiting, and diarrhea), and chemotherapy-induced peripheral neuropathy (CIPN).^20^

### Histopathology Analysis

All pathological examinations were undertaken by two experienced gastrointestinal pathologists who were blinded to the group assignment. All enrolled patients were classified as poor responders (TRG 3) based on the National Comprehensive Cancer Network (NCCN) guidelines.^21^

### Follow-Up

Patients were followed-up regularly via physical examination, radiological examination, endoscopic examination, and laboratory examination, or telephone call when visits were not possible. These examinations were performed quarterly during the first 2 years, then every 6 months until the fifth year. After 5 years, consultation and follow-ups occurred annually.

### Propensity Score Matching (PSM)

**Fig. 2:**
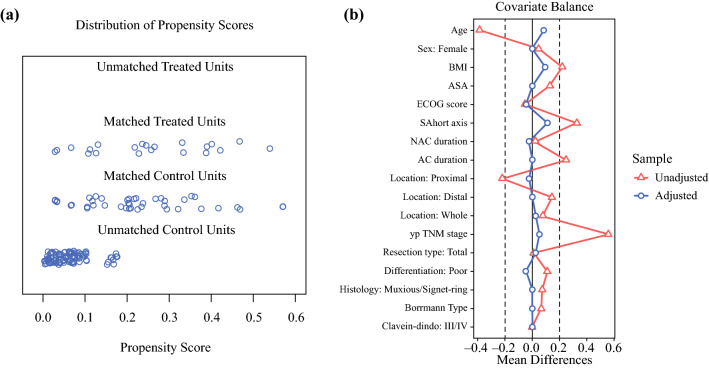
Pre-matching and post-matching information. **a** Propensity score distribution in the LTG and OTG groups before PSM application and after matching. **b** Love plot demonstrating the value of the standardized difference for each covariate included in the propensity score before and after matching. The value of the *blue circular dots* (after matching) did not exceed the absolute value of 0.20 (shown as a *dashed line*), suggesting a well-balanced distribution for all covariates after matching. *PSM* propensity score matching, *BMI* body mass index, *ECOG* Eastern Cooperative Oncology Group, *ASA* American Society of Anesthesiologists, *NAC* neoadjuvant chemotherapy, *AC* adjuvant chemotherapy

Examination of the baseline characteristics revealed significant or marginal differences in data, such as tumor size, AC duration, and tumor location. between the groups. To exclude known clinical risk factors and to calibrate the baseline, a 1:2 ratio of PSM was performed to match patients using the R ‘MatchIt’ package (R version 3.6.2; The R Foundation for Statistical Computing).^22^ All relevant confounding covariates were incorporated into the matching process, including age, BMI, sex, ASA score, ECOG score, tumor diameter, NAC duration, AC duration, tumor location, types of resection, yp TNM stage, differentiation, histological type, lymphovascular invasion (LVI), Borrmann type, and severe complications. The logit option was used to build a logistic regression model and the optimal matching option was used. To avoid overinflation and reduce variance, matching was not undertaken with replacement. A detailed patient selection method and the distribution of propensity score before and after PSM are shown in Figs. 1 and 2, respectively.

### Statistical Analysis

The independent *t*-test and the Chi-square test were used to analyze baseline differences either in the pre-match or post-match cohorts. We used a standardized mean difference (SMD) to define the matching efficacy. An SMD of <0.20 was taken as successful propensity matching between the groups.^23^ The relationships between clinical and pathological factors and long-term progression-free survival (PFS) and overall survival (OS) were assessed using univariate log-rank tests. Univariate and multivariate Cox regression analysis was applied to identify the prognostic factors of OS and PFS. Tumor or treatment characteristics that achieved a *p* value <0.10 in univariate analysis were included in the multivariate analysis. For all analyses, *p* < 0.05 was considered statistically significant. Testing for trends can be applied based on various statistical hypotheses when necessary. Statistical analyses were performed using SE STATA (Stata Statistical Software, release 15.1; Stata Corp LLC, College Station, TX, USA).

## Results

### Patient characteristics and the PSM process

The patient selection process is displayed in Fig. 1. A total of 160 patients with a TRG 3 diagnosis after more than two cycles of NAC followed by a curative gastrectomy plus D2 lymphadenectomy were identified. There were 21 patients with modified treatments, including 16 patients who changed from platinum/fluoropyrimidine to paclitaxel/fluoropyrimidine, and 5 patients who changed from paclitaxel/fluoropyrimidine to platinum/fluoropyrimidine regimens. A total of 139 patients remained on their original protocols: 132 participants were included in the platinum/fluoropyrimidine arm and 7 participants were included in the paclitaxel/fluoropyrimidine arm. The demographic and histopathological features have been summarized in Table 1, to minimize potential biases and to increase the comparability of groups.

**Table 1.** Demographic and clinicopathologic characteristics in the non-switching and switching groups before and after propensity score matching

	Unmatched cohort					Matched cohort			
	Non-switching group [*n* = 139]	Switching group [*n* = 21]	SMD	*p*-value		Non-switching group [*n* = 42]	Switching group [*n* = 21]	SMD	*p*-Value
Propensity score	0.11 ± 0.11	0.25 ± 0.15	1.070	<0.001		0.23 ± 0.14	0.25 ± 0.15	0.172	0.517
Age, years	58.82 ± 10.39	55.71 ± 8.04	0.334	0.192		55.05 ± 9.81	55.71 ± 8.04	0.074	0.789
Sex, female	33 (23.74)	6 (28.57)	0.110	0.835		12 (28.57)	6 (28.57)	<0.001	1.000
BMI, kg/m^2^	23.27 ± 3.15	24.26 ± 3.22	0.313	0.180		23.86 ± 3.22	24.26 ± 3.22	0.126	0.640
ECOG			0.254	0.700				0.103	0.924
0	92 (66.19)	15 (71.43)				28 (66.67)	15 (71.43)		
1	43 (30.94)	6 (28.57)				14 (33.33)	6 (28.57)		
2	4 (2.88)	0 (0.00)				0 (0.00)	0 (0.00)		
ASA classification			0.221	0.679				0.078	0.960
1	23 (16.55)	2 (9.57)				3 (7.14)	2 (9.52)		
2	101 (72.66)	17 (80.95)				36 (85.71)	17 (80.95)		
3	15 (10.79)	2 (9.52)				3 (7.14)	2 (9.52)		
Diameter, cm	3.29 ± 2.36	4.50 ± 3.73	0.388	0.047		4.09 ± 3.12	4.50 ± 3.73	0.119	0.640
NAC duration			0.045	1.000				0.049	1.000
2 cycles	83 (59.71)	13 (61.90)				15 (35.71)	8 (38.10)		
>2 cycles	56 (40.29)	8 (38.10)				27 (64.29)	13 (61.90)		
AC duration			0.518	0.061				<0.001	1.000
3–4 cycles	74 (53.24)	6 (28.57)				12 (28.57)	6 (28.57)		
5–6 cycles	65 (46.76)	15 (71.43)				30 (71.43)	15 (71.43)		
Tumor location			0.520	0.107				0.085	0.951
Proximal	57 (41.01)	4 (19.50)				9 (21.43)	4 (19.05)		
Distal	73 (52.52)	14 (66.67)				28 (66.67)	14 (66.67)		
Whole	9 (6.47)	3 (14.29)				5 (11.90)	3 (14.29)		
Resection			0.015	1.000				0.049	1.000
Subtotal	85 (61.15)	13 (61.90)				25 (59.52)	13 (61.90)		
Total	54 (38.85)	8 (38.10)				16 (40.48)	8 (38.10)		
ypT stage			0.317	0.737				0.112	0.915
1	5 (3.60)	0 (0.00)				0 (0.00)	0 (0.00)		
2	17 (12.23)	2 (9.52)				3 (7.14)	2 (9.52)		
3	30 (21.58)	6 (28.57)				11 (26.19)	6 (28.57)		
4	87 (62.59)	13 (61.90)				28 (66.67)	13 (61.90)		
ypN stage			0.585	0.182				0.460	0.441
0	43 (30.94)	2 (9.52)				9 (21.43)	2 (9.52)		
1	21 (15.11)	5 (23.81)				5 (11.90)	5 (23.81)		
2	34 (24.46)	5 (23.81)				12 (28.57)	5 (23.81)		
3	41 (29.50)	9 (42.86)				16 (38.10)	9 (42.86)		
yp stage			0.496	0.244				0.055	1.000
1	13 (9.35)	0 (0.00)				0 (0.00)	0 (0.00)		
2	41 (29.50)	5 (23.81)				11 (26.19)	5 (23.81)		
3	85 (61.15)	16 (76.19)				31 (73.81)	16 (76.19)		
Differentiation			0.238	0.469				0.116	0.912
Well–moderate	48 (34.53)	5 (23.81)				8 (19.05)	5 (23.81)		
Poor	91 (65.47)	16 (76.19)				34 (80.95)	16 (76.19)		
Histology			0.182	0.611				<0.001	1.000
Adenocarcinoma	116 (83.45)	16 (76.19)				32 (76.19)	16 (76.19)		
Mucinous or signet-ring	23 (16.55)	5 (23.81)				10 (23.81)	5 (23.81)		
LVI	60 (43.17)	12 (57.14)	0.282	0.335		22 (52.38)	12 (57.14)	0.096	0.929
Borrmann type			0.345	0.250				0.225	0.693
2	13 (9.35)	3 (14.29)				4 (9.52)	3 (14.29)		
3	118 (84.89)	15 (71.43)				34 (80.95)	15 (71.43)		
4	8 (5.76)	3 (14.29)				4 (9.52)	3 (14.29)		
Severe complications	20 (14.39)	3 (14.29)	0.003	1.000		6 (14.29)	3 (14.29)	<0.001	1.000

Data in parentheses are expressed as percentages

*BMI* body mass index, *ASA* American Society of Anesthesiologists, *ECOG* Eastern Cooperative Oncology Group, *NAC* neoadjuvant chemotherapy, *SMD* standardized mean difference, *LVI* lymphovascular invasion, *AC* adjuvant chemotherapy

Results from the 1:2 PSM process, as described in the Methods section, are shown in Fig. 1. The propensity score suggests there were no biases in the matched groups (0.229 ± 0.138 vs. 0.254 ± 0.148, SMD 0.172, *p* = 0.517). Following PSM, a cohort of 21 patients who switched treatment and 42 matched non-switching patients were defined (see Table 1). After the PSM protocol, we ensured that most confounders were within 0.20 SMD, except for Borrmann type (SMD 0.225, *p* = 0.693) and ypN stage (SMD 0.460, *p* = 0.441). ypN stage was not as balanced as other covariates because the yp TNM stage was regarded as the only surrogate factor for tumor staging (SMD 0.055, *p* = 1.000).

### Treatment switching effects on long-term outcomes

**Fig. 3:**
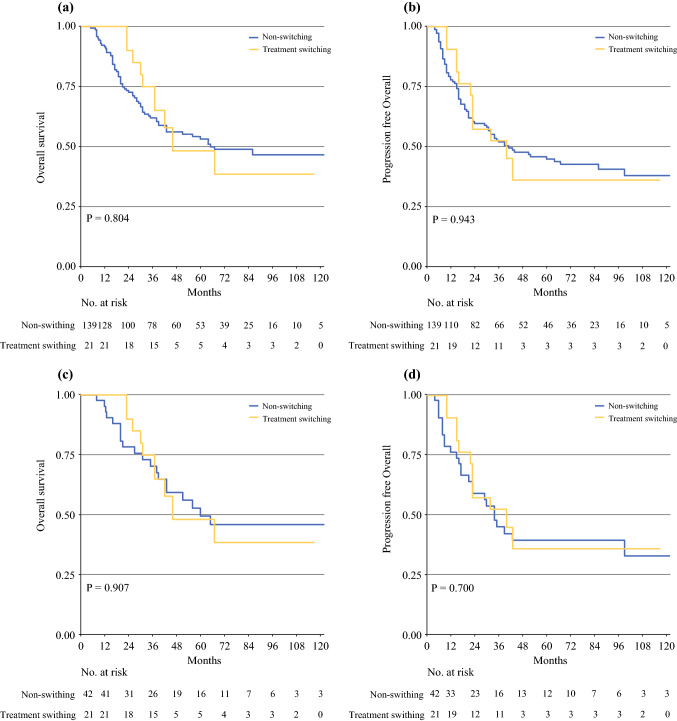
Kaplan–Meier survival plot of overall and progression-free survival before and after propensity score matching. **a, b** Survival curve of overall and progression-free survival in whole patients. **c, d** Survival curve of overall and progression-free survival after matching. Numbers at the bottom of the graphs indicate patients at risk. The *p*-value represents the log-rank test.

The median overall follow-up time was 73.3 months (45.0–99.0), with no difference between the switching and non-switching groups, either in the pre-matched cohort (switching vs. non-switching: 72 vs. 74, *p* = 0.824) or in the post-matched cohort (switching vs. non-switching: 72 vs. 73, *p* = 0.787). Comparing the survival curves for whole patients, the 5-year OS was 48.15% in the switching group and 53.08% in the non-switching group (see Fig. 3a), while the 5-year PFS was 35.92% in the switching group and 44.75% in the non-switching group (Fig. 3b). There was no significant difference in either OS (log-rank *p* = 0.804) or PFS (log-rank *p* = 0.943). Similarly, within the post-matched cohort, switching treatment was associated with no significantly different outcomes compared with the non-switching group (OS: log-rank *p* = 0.907; PFS: log-rank *p* = 0.670), as shown in Fig. 3c and d.

**Fig. 4:**
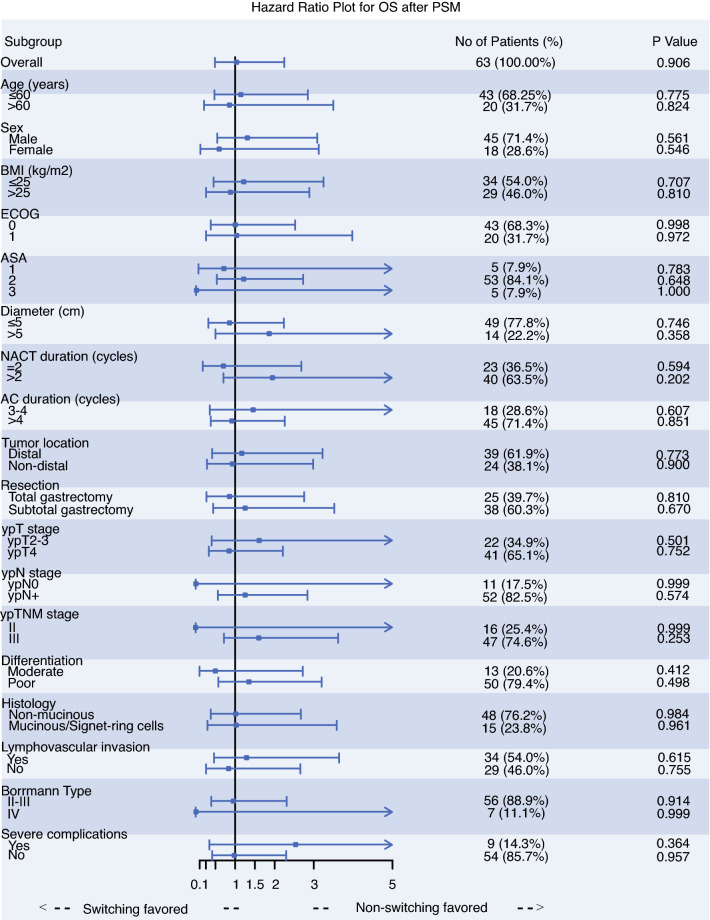

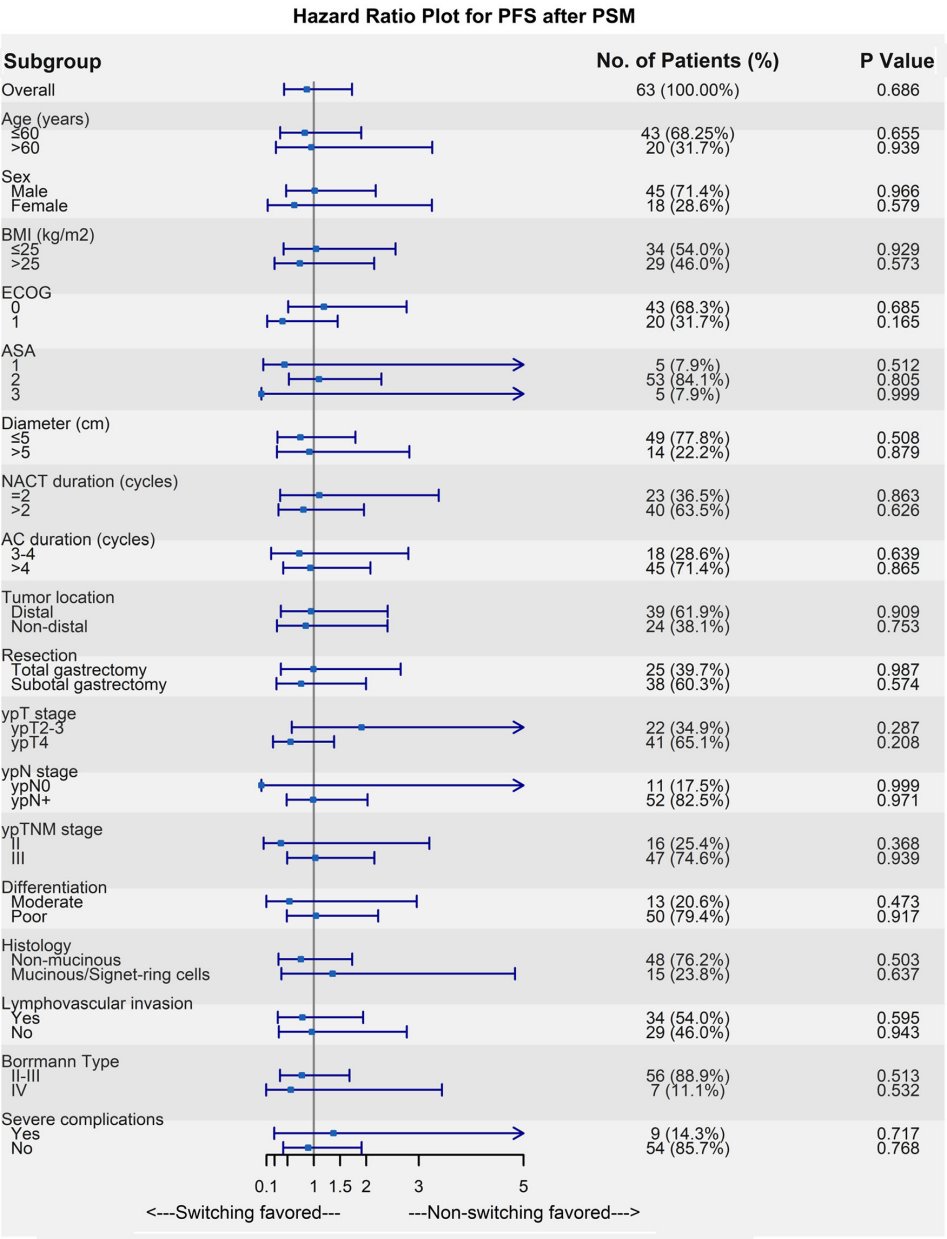
Subgroup analysis of **a** OS in the matched cohort, and **b** PFS in the matched cohort. *OS* overall survival, *PSM* propensity score matching, *PFS* progression-free survival, *BMI* body mass index, *ECOG* Eastern Cooperative Oncology Group, *ASA* American Society of Anesthesiologists, *NACT* neoadjuvant chemotherapy, *AC* adjuvant chemotherapy

An exploratory subgroup analysis was conducted to analyze the survival benefit in the cohort before and after PSM, respectively (electronic supplementary Fig. S1; Fig. 4). The OS and PFS outcomes were comparable between the switching and non-switching groups in most subgroups. A significant survival benefit could only be found in the ypT<4 subgroup among the whole patients (electronic supplementary Fig. S1b), in which the 5-year PFS was 18.75% in the switching group, compared with 68.86% in the non-switching group (hazard ratio [HR] 3.28, 95% confidence interval [CI] 1.25–8.56; *p* = 0.016). In the corresponding PSM cohort, the 5-year PFS was 63.49% in the non-switching group, with no statistical significance between groups (HR 1.91, 95% CI 0.58–6.28; *p* = 0.287). Kaplan–Meier plots of OS and PFS for ypT<4 patients before and after PSM are presented in electronic supplementary Fig. S2.

### Risk Factors for Patient Survival

The prognostic value of all the recorded clinicopathological factors was evaluated using univariate and multivariate Cox regression analysis. Among 160 patients, the univariate analysis indicated that the variables of tumor diameter >5 cm, non-distal location, total gastrectomy, advanced ypTNM stage, poorer histological subtype, LVI, and Borrmann type IV were correlated with an unfavorable prognosis, both for OS and PFS, while those with higher ECOG score were correlated only for PFS (see Table 2). When candidate prognostic factors were subjected to multivariate models, only ypTNM stage was identified as an independent prognostic factor in both OS (III vs. I/II: HR 3.04, 95% CI 1.56–5.94; *p* = 0.001) and PFS (III vs. I/II: HR 2.85, 95% CI 1.52–5.33; *p* = 0.001). On the other hand, Bormann type IV independently predicted poorer OS (HR 3.51, 95% CI 1.73–7.11; *p* = 0.043) but not PFS (HR 1.64, 95% CI 0.72–3.73; *p* = 0.238) [see Table 2].

**Table 2.** Univariate and multivariate analyses of prognostic factors

	OS				PFS			
	Univariate HR (95% CI)	*p*-value	Multivariate HR (95% CI)	*p*-value	Univariate HR (95% CI)	*p*-value	Multivariate HR (95% CI)	*p*-value
Age >60 years	1.43 (0.91–2.23)	0.121			1.33 (0.88–2.01)	0.182		
Sex, female	1.16 (0.69–1.95)	0.570			0.84 (0.51–1.39)	0.497		
BMI >25 kg/m^2^	0.70 (0.43–1.16)	0.166			0.70 (0.44–1.11)	0.131		
ECOG >0	1.36 (0.86–2.15)	0.189			1.45 (0.95–2.21)	0.086	1.32 (0.83–2.10)	0.237
ASA classification								
1	1.00							
2	0.89 (0.49–1.64)	0.717			1.09 (0.60–1.99)	0.760		
3	0.87 (0.37–2.03)	0.744			0.98 (0.43–2.24)	0.961		
Diameter, cm	2.74 (1.59–4.72)	<0.001	1.24 (0.65–2.37)	0.507	3.45 (2.07–5.78)	<0.001	1.70 (0.93–3.11)	0.082
NAC duration, >2 cycles	1.26 (0.79–2.01)	0.334			1.13 (0.74–1.73)	0.568		
AC duration, >4 cycles	0.85 (0.54–1.32)	0.463			0.92 (0.61v1.39)	0.684		
Tumor location, distal vs. others	1.87 (1.19–2.95)	0.006	1.37 (0.74–2.56)	0.316			1.53 (0.88–2.67)	0.131
Total gastrectomy	1.88 (1.20–2.95)	0.006	1.19 (0.62–2.29)	0.603	2.09 (1.38–3.16)	0.001	1.11 (0.62–1.98)	0.731
ypT4 stage	2.16 (1.27–3.66)	0.004			2.22 (1.37–3.59)	0.001		
ypN+ stage	3.01 (1.62–5.58)	<0.001			3.53 (1.95–6.37)	<0.001		
ypTNM stage III	3.77 (2.11–6.75)	<0.001	3.04 (1.56–5.94)	0.001	4.26 (2.47–7.34)	<0.001	2.85 (1.52–5.33)	0.001
Poor differentiation	0.98 (0.61–1.57)	0.933			0.363 (0.79–1.92)	0.363		
Mucinous or signet-ring cells	1.74 (1.02–2.95)	0.041	1.32 (0.75–2.33)	0.337	1.59 (0.97–2.62)	0.068	1.30 (0.76–2.22)	0.338
LVI	2.13 (1.35–3.35)	0.001	1.09 (0.64–1.88)	0.742	2.60 (1.70–3.99)	<0.001	1.45 (0.88–2.40)	0.148
Borrmann type IV	3.51 (1.73–7.11)	<0.001	2.40 (1.03–5.60)	0.043	2.96 (1.47–5.97)	0.002	1.64 (0.72–3.73)	0.238
Severe complications	1.54 (0.87–2.70)	0.135			1.43 (0.84–2.43)	0.182		
Switching treatment	0.92 (0.47–1.79)	0.805			0.98 (0.53–1.80)	0.944		

*BMI* body mass index, *ASA* American Society of Anesthesiologists, *ECOG* Eastern Cooperative Oncology Group, *HR* hazard ratio, *PFS* progression-free survival, *OS* overall survival, *NAC* neoadjuvant chemotherapy, *CI* confidence interval, *AC* adjuvant chemotherapy, *LVI* lymphovascular invasion

### Adverse Events

The major AEs recorded during chemotherapy for the matched cohort are listed in Table 3. No deaths related to chemotherapy were observed. The overall rate of AEs was 95.24% in the non-switching group, marginally higher than the 80.95% rate in the switching group (*p* = 0.069). Nevertheless, the grade 3/4 AE rates were comparable in the two groups (non-switching vs. switching: 30.95% vs. 28.57%, *p* = 0.846). Although incidences between the two matched groups were comparable, in each AE subclass there were three tiers, including leukopenia, anemia, thrombocytopenia, hepatotoxicity, digestive tract disorders, and neurotoxicity. The global rate of neurotoxicity was found to be significantly higher in treatment-switched patients (non-switchers vs. switchers: 19.05% vs. 42.86%, *p* = 0.045). On the other hand, there was no significant difference in the frequency of any types of AEs that occurred in the whole patients, whether stratified by CTCAE or not (Table 4).

**Table 3.** Adverse events of adjuvant chemotherapy in patients in the switching or non-switching treatment groups after propensity score matching, based on CTCAE v4.0

Adverse events	Non-switching group [*n* = 42] (%)	Switching group [*n* = 21] (%)	*p*-value (grade 0 vs. >0)	*p*-value^a^
Leukopenia			0.160	0.279
Grade 0	9 (21.43)	8 (38.10)		
Grade 1/2	21 (50.00)	8 (38.10)		
Grade 3/4	12 (28.57)	5 (23.81)		
Anemia			0.711	0.714
Grade 0	26 (61.90)	14 (66.67)		
Grade 1/2	16 (38.10)	7 (33.33)		
Grade 3/4	0 (0.00)	0 (0.00)		
Thrombocytopenia			1.000	1.000
Grade 0	36 (85.71)	18 (85.71)		
Grade 1/2	6 (14.29)	3 (14.29)		
Grade 3/4	0 (0.00)	0 (0.00)		
Hepatotoxicity			0.466	0.470
Grade 0	24 (57.14)	14 (66.67)		
Grade 1/2	18 (42.86)	7 (33.33)		
Grade 3/4	0 (0.00)	0 (0.00)		
Digestive tract disorders			0.475	0.478
Grade 0	18 (42.86)	11 (52.38)		
Grade 1/2	24 (57.14)	10 (47.62)		
Grade 3/4	0 (0.00)	0 (0.00)		
Neurotoxicity			0.045	0.063
Grade 0	34 (80.95)	12 (57.14)		
Grade 1/2	7 (16.67)	8 (38.10)		
Grade 3/4	1 (2.38)	1 (4.76)		
Adverse outcomes, in total			0.069	0.299
Grade 0	2 (4.76)	4 (19.05)		
Grade 1/2	27 (64.29)	11 (52.38)		
Grade 3/4	13 (30.95)	6 (28.57)		

*CTCAE* Common Terminology Criteria for Adverse Events

^a^Chi-square test for linear trend applied across ordered categories

**Table 4.** Adverse events of adjuvant chemotherapy in patients in the switching or non-switching treatment groups (in whole patients), based on CTCAE v4.0

Adverse events	Non-switching group [*n* = 139] (%)	Switching group [*n* = 21] (%)	*p*-value (grade 0 vs. >0)	*p*-value^a^
Leukopenia			0.310	0.529
Grade 0	38 (27.34)	8 (38.10)		
Grade 1/2	68 (48.92)	8 (38.10)		
Grade 3/4	33 (23.74)	5 (23.81)		
Anemia			0.773	0.773
Grade 0	97 (69.78)	14 (66.67)		
Grade 1/2	42 (30.22)	7 (33.33)		
Grade 3/4	0 (0.00)	0 (0.00)		
Thrombocytopenia			0.521	0.202
Grade 0	102 (73.38)	18 (85.71)		
Grade 1/2	34 (24.46)	3 (14.29)		
Grade 3/4	3 (2.16)	0 (0.00)		
Hepatotoxicity			0.429	0.430
Grade 0	104 (74.82)	14 (66.67)		
Grade 1/2	35 (25.18)	7 (33.33)		
Grade 3/4	0 (0.00)	0 (0.00)		
Digestive tract disorders			0.149	0.120
Grade 0	50 (35.97)	11 (52.38)		
Grade 1/2	85 (61.15)	10 (47.62)		
Grade 3/4	4 (2.88)	0 (0.00)		
Neurotoxicity			0.168	0.101
Grade 0	100 (71.94)	12 (57.14)		
Grade 1/2	38 (27.34)	8 (38.10)		
Grade 3/4	1 (0.72)	1 (4.76)		
Adverse outcomes in total			0.138	0.419
Grade 0	12 (8.63)	4 (19.05)		
Grade 1/2	86 (61.87)	11 (52.38)		
Grade 3/4	41 (29.50)	6 (28.57)		

*CTCAE* common terminology criteria for adverse events

^a^Chi-square test for linear trend applied across ordered categories

## Discussion

Following the conclusion of the MAGIC trial, with the accumulation of clinical benefit data,^2^,^11^,^24^,^25^ PEC gradually became standardized in Western countries and parts of East Asia.^13^–^15^ Despite the lack of large-scale phase III clinical research in China, the perioperative treatment modality revealed a prominent interim outcome in the RESOLVE trial (NCT01534546), with the perioperative SOX arm shown to be superior to the postoperative XELOX arm in 3-year disease-free survival (DFS) in LAGC patients undergoing D2 gastrectomy.^26^ Even though preoperative chemotherapy is responsive and more tolerable in most LAGC patients, and can enhance the resectability,^5^ approximately one-third of patients still tend to manifest poor or even no response after NAC, as measured using different TRG systems.^9^,^10^,^27^ Furthermore, there are still concerns as to whether the TRG can accurately reflect the NAC efficacies, as the shrinkage of primary tumor bed may occur simultaneously, challenging the identification of the original tumorous borders and volumes.^28^ These unresolved outcomes make the postoperative treatment of patients with a poor response following NAC difficult to predict—almost a ‘trial and error’ approach.

Intuitively, a poor TRG, or little regression in the pathologic specimen, would suggest ineffective neoadjuvant treatment. It is theoretically posited that NAC enables monitoring chemotherapy responses at an early stage, potentially conferring time and flexibility to switch therapies for poor responders; however, as selections of first-line therapies for LAGC are scarce, this strategy does not provide much of an advantage. Only fluorouracil plus platinum- and/or taxane-based regimens have been proven to be effective in PEC settings according to ESMO and NCCN guidelines.^14^,^15^ That is, if a patient has received 5-fluorouracil/leucovorin, oxaliplatin, docetaxel (FLOT) as preoperative treatment, then any non-cross-resistant chemotherapy is currently unwarranted.

Wang et al. retrospectively reviewed 12 patients with treatment modifications (from oxaliplatin to taxane-based protocols), compared with 24 deliberately matched control cases who retained the original treatment protocols. All patients received an oxaliplatin plus 5-fluorouracil-based regimen as preoperative treatment but were reported to show minimal responses for NAC, with a graded histologic regression (GHR) <50%. The OS outcomes were not significantly improved in the modified group. Although the study tested the hypothesis that poor responders with stage III LGAC (AJCC 7th edition) are a potentially beneficial group for changing treatments, the data did not confirm this outcome. No further studies with larger sample sizes have committed to confirm this hypothesis as yet.

However, in the current study, the treatment-switched strategy showed no statistically significant survival improvement, either in whole patients or in a stringent PSM cohort. The subgroup analysis, except for the ypT<4 category, did not reveal any merit or disadvantages in treatment switching. The tolerance of treatment switching was globally comparable between the two treating strategies, although the derived marginal significance (*p* = 0.069) might deserve further investigations as the treatment duration has been well-matched in the PSM cohort. In contrast, the rate of CIPN was significantly higher in the treatment-switched group after PSM. This result could be because 62% of patients in the treatment-switched group received oxaliplatin/docetaxel sequential therapy. Although CIPN patterns do share similarities, it is known that recovery from CIPN is delayed in oxaliplatin compared with paclitaxel, always requiring 2–3 months following cessation of therapy.^29^ The recovery period overlapped with the timeline between the completion of NAC and the initiation of AC. The coincident timing window could have resulted in treatment-switched patients being treated with two types of neurotoxic agents.^30^

As for the ypT<4 subclass, we found a global strength of PFS benefit in non-switching patients before PSM. Although this statistical significance did not persist in PFS after PSM and in the OS analysis, a clear trend of survival inferiority can be found in the switching group, with only 26.79% 5-year OS and 18.75% 5-year PFS (electronic supplementary Fig. S2). In these eight patients, comprising of five patients with ypN3 status (including four patients with ypN3b status and one patient with ypN3a status), all ypN3 (62.50%) patients confronted recurrence and all four N3b (50.00%) patients suffered death due to tumor metastasis. Compared with the non-switching group, with only 17.31% of ypN3 patients in the ypT<4 subcategory, there was a significantly higher proportion of ypN3 patients in the switching group (62.50% vs. 17.31%, *p* = 0.005). As the size of the subgroup was quite small, the influence of the confounders cannot be neglected. Therefore, the unequal distribution of ypN status should be the most likely reason for these survival differences in the ypT<4 subgroup because the lymph node metastasis is most prognostic for gastric cancer, as advocated by the current dataset (Table 2). Nonetheless, all our results obtained thus far showed that a second non-cross-resistant regimen such as AC did not result in either a better survival outcome or increased tolerance in NAC poor responders.

Similarly, in breast cancer (BC), von Minckwitz et al. investigated clinical outcomes when switching to non-cross-resistant chemotherapy in patients who did not respond to the initial induced chemotherapy. The interim evaluation was performed by sonography after two cycles of TAC (docetaxel, doxorubicin, and cyclophosphamide). Patients with no change in tumor size after the initial treatment were randomized to (1) use an additional four cycles of TAC, or (2) switch to a non-cross-resistant NX regimen (vinorelbine and capecitabine). The final pathological response showed no significant difference between these two treatment protocols, but fewer toxic effects were observed in the treatment switch group, unlike ultrasonography for BC, which proved to have satisfactory diagnostic accuracy in superficial soft tissue tumors. Current diagnostic efficacies in post-treatment assessment for LAGC, either CT or EUS, show that baseline gastrointestinal tract tumors are often unmeasurable according to the Response Evaluation Criteria in Solid Tumors (RECIST) criteria, resulting in insufficiently quantified comparisons in many studies.^31^–^33^ Consequently, there is currently a lack of consensus on reliable post-treatment evaluations for LAGC in the absence of pathology data.^34^ Moreover, unlike BC, which has a relatively better grade of differentiation and slower metastasis tendencies, gastrointestinal tumors are more invasive and are not always chemosensitive. As a result, timely surgery for resectable LAGC is an important factor in managing patients’ survival outcomes.^35^ Therefore, treatment using NAC is undertaken with greater caution in LAGC patients as D2 surgery presents better outcome opportunities and consideration of alternative chemotherapy protocols occurs post-surgery. More recently, in the field of treating BC, results from the CREATE-X trial showed that the addition of capecitabine has a statistically significant survival advantage in human epidermal growth factor receptor 2 (HER2)-negative residual invasive BC patients with no pathologic complete response after NAC. In the subgroup analysis within that study, patients who did not use 5-fluorouracil as an NAC regimen had a statistical benefit in DFS with the inclusion of capecitabine, based on original postoperative protocols (capecitabine group vs. control: HR 0.63, 95% CI 0.63–0.99).^36^ The successful design of the CREATE-X trial was due to the discriminatory molecular-based subtyping method, where patients with potential benefit were enriched due to the wide range and number of regimen selections for treating BC.

Although the treatment of LAGC has evolved into the era of precision medicine, chemotherapy is still the principal strategy in preoperative clinical settings, simply because there is currently no evidence to support the use of biologically targeted drugs and anti-angiogenic compounds that can statistically improve patients’ survival prognosis.^37^,^38^ The effectiveness of molecular classifications for GC still needs to be validated in large, population-based, prospective datasets.^39^ Nonetheless, according to The Cancer Genome Atlas (TCGA) molecular subclassification, patients with mismatch repair (MMR) deficiency or microsatellite high instability (MSI-H) status show a better prognosis but lower chemosensitivity compared with MSI-low (MSS) patients.^40^ This tentative conclusion has been confirmed by post hoc analysis of data from the MAGIC and CLASSIC trials.^41^,^42^ The consensus of current experience on MSI (and other TCGA subtypes) provides significant, important guidelines for treating post-NAC poor responders. Not only does it help to avoid unnecessary chemotherapy but it also offers a cost-effective clinical strategy for those patients unable to afford the high price of genetic testing, especially for the first round of chemotherapy.^43^ Importantly, the current study indicates that a treatment-switched strategy based only on a histopathological assessment is not a valid protocol for non-responders.

As NAC and PEC for LAGC have become widespread across the Western world and are gradually being popularized in East Asia, patients who do not respond to chemotherapy remain a challenge in LAGC treatment. These patients may potentially have their treatments augmented by linking with specific histological or molecular subclassifications. To investigate whether poorly responsive patients can benefit from changing treatments or including additional agents (supplemental to the original plan), a prospective study that ‘enriches’ the intent-to-treat population is necessary so that we can learn from the success of the CREATE-X trial.^44^

Some limitations of this study need to be considered. First, our study was limited by the retrospective nature of the analysis, and all confounding factors could not be varied or controlled. The application of stringent selection criteria, together with a PSM method to minimize the systemic and statistical bias, were two compensatory factors employed to overcome this inherent limitation. Second, our treatment-switched group was restricted to interchanging between paclitaxel and oxaliplatin in dual-drug protocols. Additional chemotherapy agents were consequently not included; for example, from doublet XELOX to triplet FLOT, and similar sequential management protocols. The number of patients with additional chemotherapy in our initial dataset is quite small, all of whom either did not complete sufficient cycles of treatment or due to other excluded reasons. Third, the threshold for switching treatments was based on evaluations using the AJCC/CAP TRG system. As miscellaneous TRG systems for gastric cancer are available globally, derived with different principles, different layers, and different cut-off values, whether the recommended AJCC/CAP system by the Chinese Society of Clinical Oncology is valid to ascertain those patients who need a treatment switch needs further investigation.^13^

## Conclusion

The results from the current study demonstrate that within the guidelines for recommended chemotherapeutic drugs in a perioperative setting for LAGC, treatment switch strategies based on a post-NAC pathological evaluation offer no benefits, either in short-term tolerance or long-term survival, in comparison with patients who did not switch. However, these conclusions warrant further investigations, using a larger sample database, together with a finer selection of patients using non-cross-resistant regimens as post-NAC treatment in any future prospective studies.

## Supplementary Information

Below is the link to the electronic supplementary material.

**SUPPLEMENTARY FIG. S1** Subgroup analysis of (a) overall survival in the entire cohort, and (b) progression-free survival in the entire cohort. **SUPPLEMENTARY FIG. S2** Kaplan–Meier survival plot of overall survival (OS) and progression-free survival (PFS) before and after PSM in the ypT<4 subclass. Survival curve of OS and PFS (a, b) in whole patients, and (c, d) after matching. Numbers at the bottom indicate patients at risk. The p-value represents the log-rank test. (ZIP 1769 kb)

## Data Sharing Statement

The raw data supporting the conclusions of this article will be made available by the authors upon reasonable request. Requests to access the datasets should be directed to Ziyu Li (ziyu_li@hsc.pku.edu.cn).
