# Supplementary figures and images for: Treatment Switch in Poor Responders with Locally Advanced Gastric Cancer After Neoadjuvant Chemotherapy
Source: Ann Surg Oncol. 2021 Jul 29;28(13):8892–907. doi: 10.1245/s10434-021-10087-x (PMC8591025; doi:10.1245/s10434-021-10087-x)

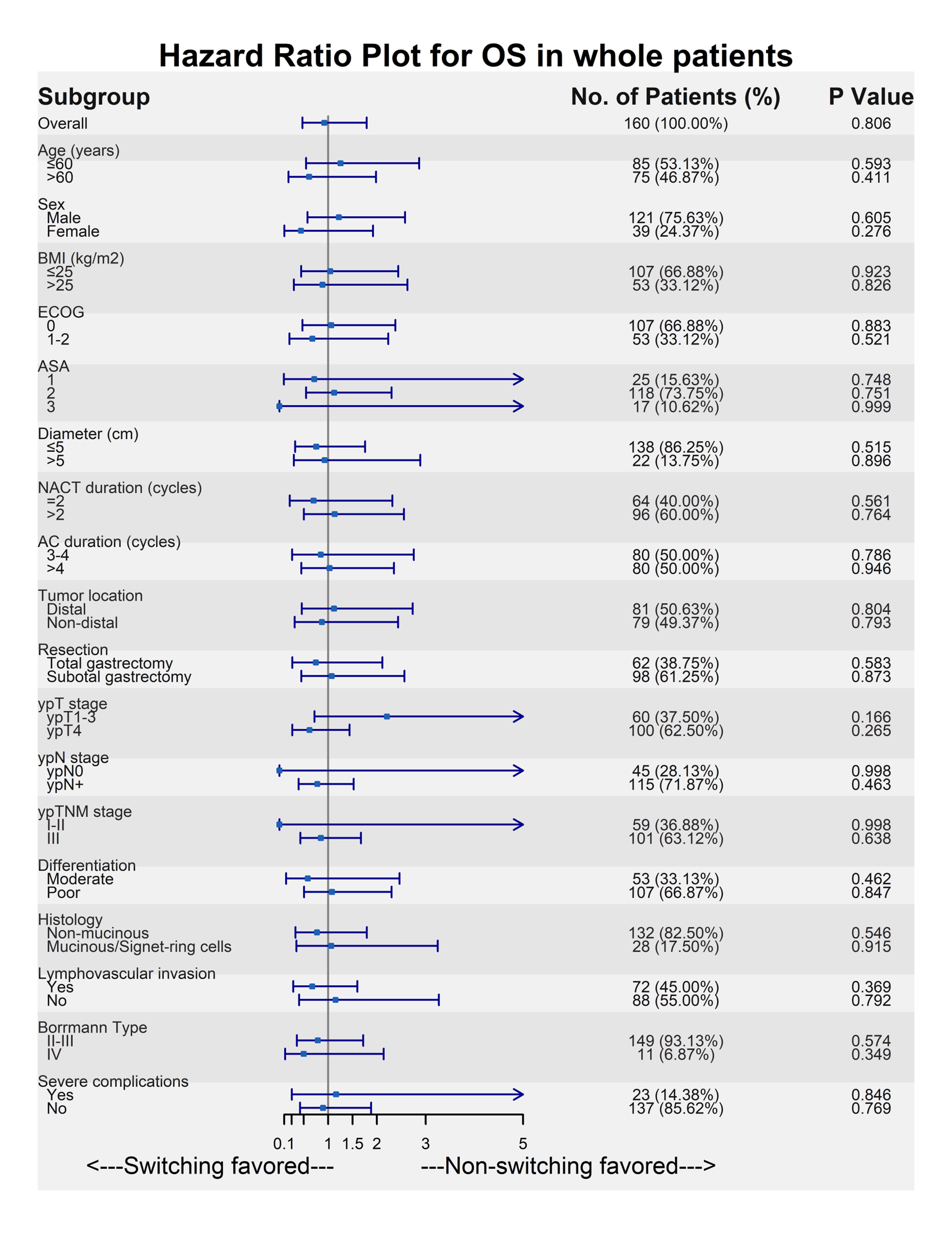

Supplement: Supplementary file 1 — SUPPLEMENTARY FIG. S1 Subgroup analysis of (a) overall survival in the entire cohort, and (b) progression-free survival in the entire cohort. SUPPLEMENTARY FIG. S2 Kaplan–Meier survival plot of overall survival (OS) and progression-free survival (PFS) before and after PSM in the ypT<4 subclass. Survival curve of OS and PFS (a, b) in whole patients, and (c, d) after matching. Numbers at the bottom indicate patients at risk. The p-value represents the log-rank test. (ZIP 1769 kb) [file 10434_2021_10087_MOESM1_ESM.zip › 10434_2021_10087_MOESM1_ESM/aso-2021-01-0153-File007.jpg]

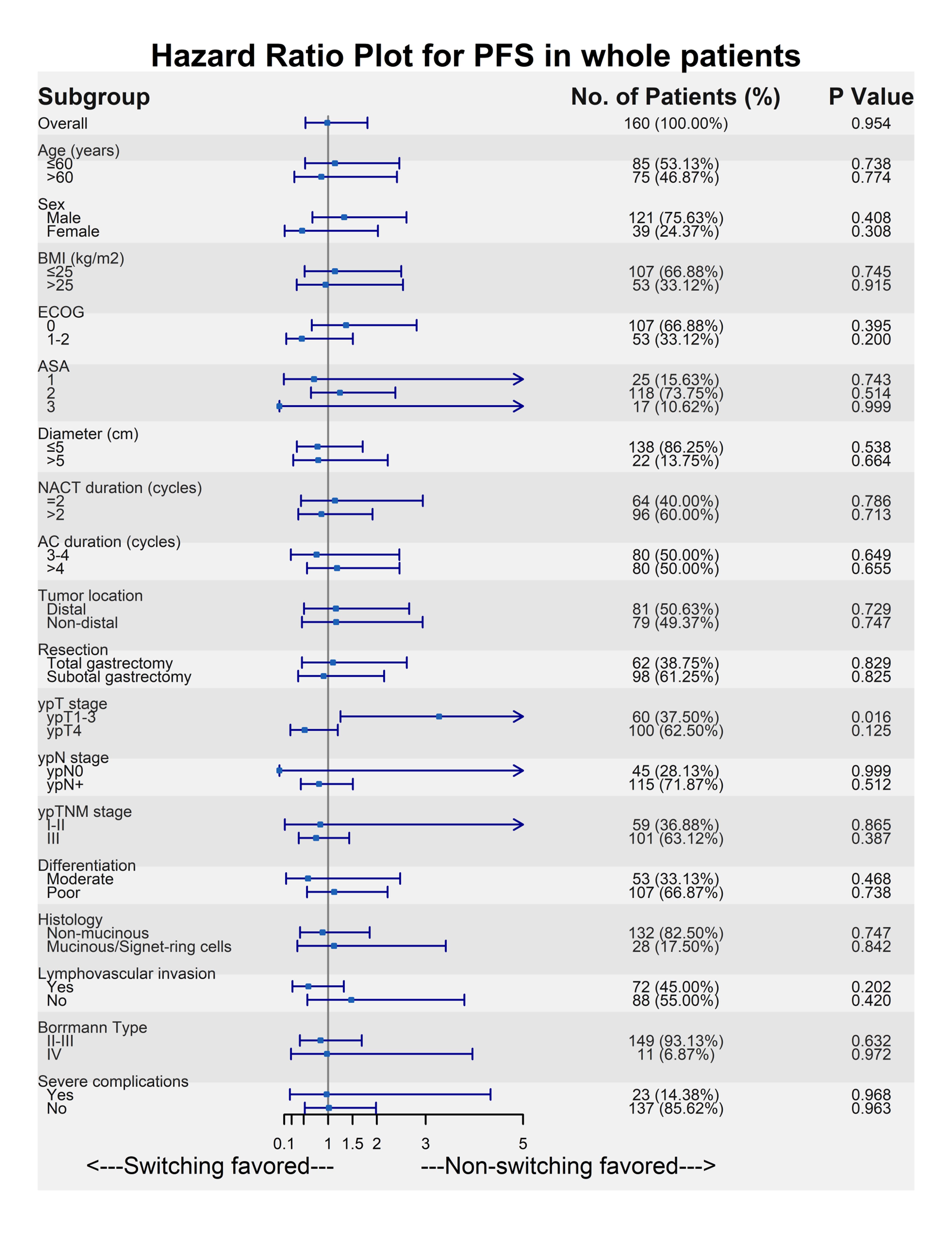

Supplement: Supplementary file 1 — SUPPLEMENTARY FIG. S1 Subgroup analysis of (a) overall survival in the entire cohort, and (b) progression-free survival in the entire cohort. SUPPLEMENTARY FIG. S2 Kaplan–Meier survival plot of overall survival (OS) and progression-free survival (PFS) before and after PSM in the ypT<4 subclass. Survival curve of OS and PFS (a, b) in whole patients, and (c, d) after matching. Numbers at the bottom indicate patients at risk. The p-value represents the log-rank test. (ZIP 1769 kb) [file 10434_2021_10087_MOESM1_ESM.zip › 10434_2021_10087_MOESM1_ESM/aso-2021-01-0153-File008.jpg]

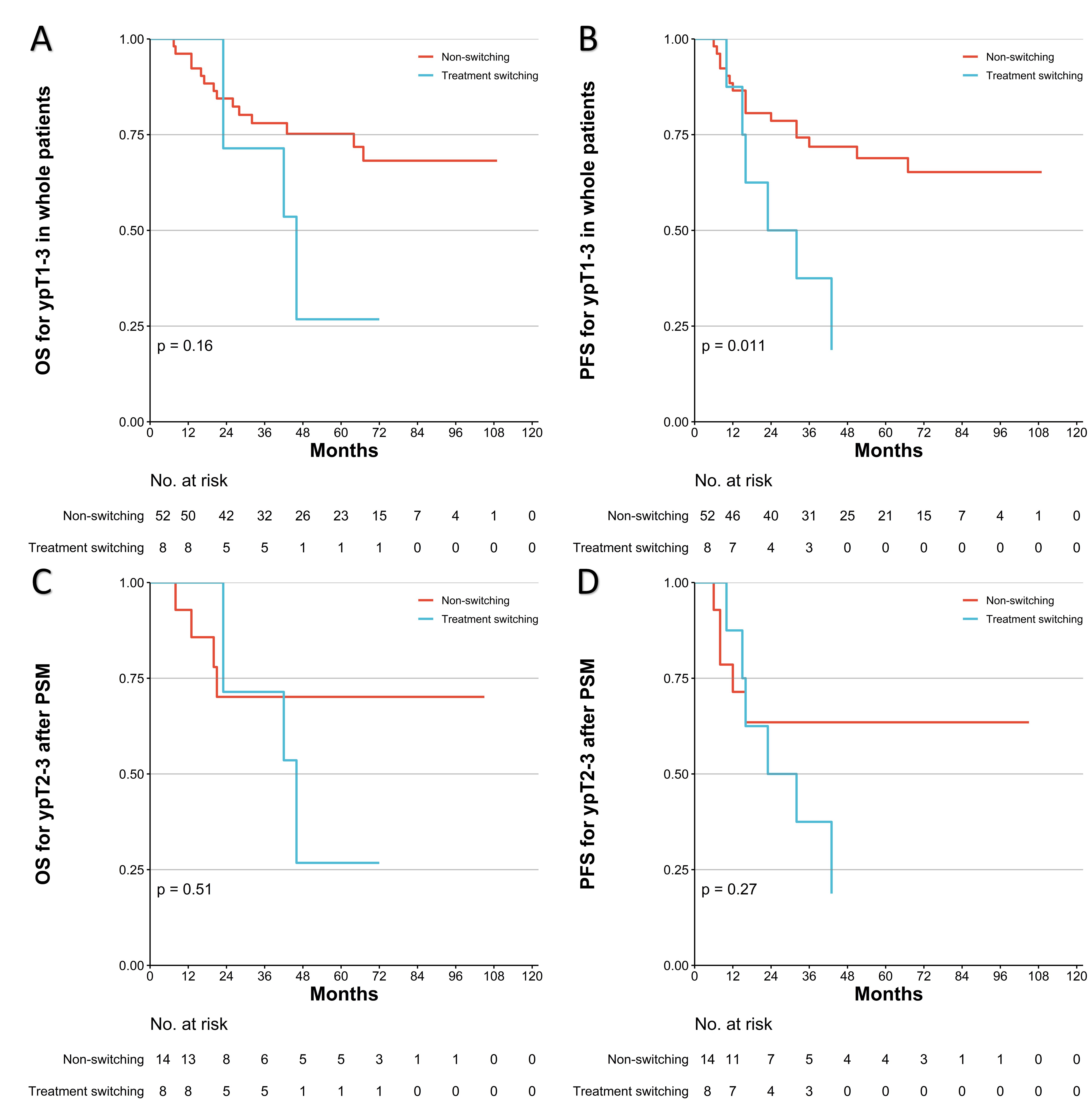

Supplement: Supplementary file 1 — SUPPLEMENTARY FIG. S1 Subgroup analysis of (a) overall survival in the entire cohort, and (b) progression-free survival in the entire cohort. SUPPLEMENTARY FIG. S2 Kaplan–Meier survival plot of overall survival (OS) and progression-free survival (PFS) before and after PSM in the ypT<4 subclass. Survival curve of OS and PFS (a, b) in whole patients, and (c, d) after matching. Numbers at the bottom indicate patients at risk. The p-value represents the log-rank test. (ZIP 1769 kb) [file 10434_2021_10087_MOESM1_ESM.zip › 10434_2021_10087_MOESM1_ESM/aso-2021-01-0153-File009.jpg]
